# Human Papillomavirus-Negative Cervical Cancer: A Comprehensive Review

**DOI:** 10.3389/fonc.2020.606335

**Published:** 2021-02-17

**Authors:** Biyuan Xing, Jianfeng Guo, Yuhan Sheng, Gang Wu, Yingchao Zhao

**Affiliations:** ^1^ Cancer Center, Union Hospital, Tongji Medical College, Huazhong University of Science and Technology, Wuhan, China; ^2^ Department of Obstetrics and Gynecology, Union Hospital, Tongji Medical College, Huazhong University of Science and Technology, Wuhan, China

**Keywords:** human papillomavirus, human papillomavirus (HPV) testing, false-negative, human papillomavirus (HPV)-negative, cervical cancer

## Abstract

Human papillomavirus (HPV) has been the leading cause of cervical cancer for over 25 years. Approximately 5.5–11% of all cervical cancers are reported to be HPV-negative, which can be attributed to truly negative and false-negative results. The truly HPV-negative cervical cancers are almost all cervical adenocarcinomas with unclear etiology. False HPV negativity can arise from histological misclassification, latent HPV infection, disruption of the targeting fragment, non-high risk HPV infection, and HPV testing methods. HPV-negative cervical cancers are often diagnosed at an advanced FIGO stage and have a poor prognosis; thus, the management of these cases requires greater attention.

## Introduction

Cervical cancer is the fourth most common malignancy among women worldwide, accounting for approximately 7% of all cancer cases in women (1, 2). Persistent infection with human papillomavirus (HPV), particularly high-risk genotypes of HPV, is considered the major cause of cervical cancer. HPV DNA replicates from free DNA in the basal cells of the cervix during the initial period of HPV infection, and then integrates into the host genome as the infection progresses, with subsequent upregulation of E6 and E7 oncogene expression (3). HPV can be found in almost all cervical squamous cell carcinomas and precancerous lesions, including high grade squamous intraepithelial lesions (HSILs) or grade 2–3 cervical intraepithelial neoplasia (CIN). Although the sensitivity of HPV testing has improved significantly in recent years, a small fraction of cervical cancers are continued to be reported as HPV-negative. HPV-negative cervical cancer is often diagnosed at an advanced FIGO stage and associated with poor prognosis. Insights into the etiology, therapy, and prognosis of HPV-negative cervical cancer may help develop appropriate strategies for its management in patients.

Currently, there is no clear definition of HPV-negative cervical cancer to describe cases diagnosed by pathological features in the absence of HPV-infection *via* HPV testing. The existence of cervical adenocarcinoma independent of HPV infection has been recognized by the majority of researchers (4–6). It is estimated that approximately 5.5–11% of cervical cancers worldwide are HPV-negative (7–10). A review of 243 studies and 30,848 women with invasive cervical cancer that were reported between 1990 and 2010 revealed a gradual decrease in the number of HPV-negative cases (11). In this meta-analysis, the incidence of HPV-positivity in 1990–1999, 2000–2005, and 2006–2010 was 85.9%, 87.9%, and 92.9%, respectively. The downward trend in HPV-negativity could be related to improvements in HPV testing and non-cervical cancer classification. A recent study using next-generation sequencing (NGS) to characterize primary cervical cancer revealed that HPV-negative cervical cancer accounted for approximately 5% of all cervical cancer cases (12). However, only a few rare pathological types of cervical cancer are truly HPV-negative (13–15). In studies involving HPV testing, the true incidence of HPV-negative invasive cervical cancer might be overestimated (16).

This review provides a comprehensive overview of the attributable reasons, clinical characteristics, treatment, and prognostic measures for HPV-negative cervical cancer, with the aim to assist in the development of effective therapeutic strategies to improve clinical outcomes.

## Human Papillomavirus-Negative Cervical Cancer: Attributable Reasons

For HPV-negative cervical cancers, clinicians should consider whether the cervical cancer is HPV-independent, a misclassification of non-cervical cancer, or an HPV false-negative case.

### Human Papillomavirus-Independent Cervical Cancer

HPV-independent cervical cancer, considered to be “truly” HPV-negative, is not associated with HPV infection. Cervical squamous cell carcinoma is rarely HPV-negative (17), and a confirmed HPV-independent cervical squamous cell carcinoma has not yet been reported. For cervical adenocarcinoma, the HPV negativity rate is approximately 15–38% (10, 18, 19). The HPV positivity rate in carcinoma *in situ* varies according to different histological features (17). Although the exact mechanism underlying HPV-independent cervical cancer is unclear, most researchers consider it to be caused by mutations in tumor-associated genes such as TP53, PIK3CA, and CDKN2A (20).

### Misclassification of Non-Cervical Cancer

Cervical cancers include the direct extension of endometrial carcinoma or those arising from distant metastasis of other primary HPV-negative tumors. Research has shown that almost 68% of HPV-negative cervical cancers were misdiagnosed as primary cervical cancer (7). A study examining HPV-negative cervical adenocarcinoma indicated that more than 50% of cases could not be distinguished from endometrial carcinoma based on histological features alone (21). Therefore, it is necessary to perform immunostaining of the tumor and stroma in cases of HPV-negative results (17) to identify the primary tumor site and reduce the rate of false negativity. A combination of estrogen receptor (ER), progesterone receptor (PR), vimentin, and CD10 negativity along with carcinoembryonic antigen (CEA), diffuse p16, CD34, and HPV positivity suggests cervical adenocarcinoma, while a combination of ER, PR, vimentin, diffuse p16, and CD10 positivity along with CEA, CD34, and HPV negativity suggests uterine adenocarcinoma (17, 22). Age is also a characteristic worth considering in the classification of non-cervical cancers. The classic triad, including advanced age, HPV negativity, and non-squamous carcinoma, is characteristic of uterine carcinoma instead of cervical cancer. Compared with other sites, such as the gynecologic tract, it is rare for the uterine cervix to be a metastatic site considering its anatomy (23); however, 3.7% of female genital metastatic tumors reportedly involve the uterine cervix (24).

### Human Papillomavirus False-Negative Cervical Cancer

#### Latent Human Papillomavirus Infection

Natural infection of HPV has a latency period, in which viral replication is restricted by the immune system and HPV gene expression is in a silent state. However, the natural history of HPV from infection to cervical cancer remains unclear (25). A 5-year follow-up study involving sensitive HPV DNA testing revealed that most HPV infections disappeared within two years, except those with precancerous lesions or worse (26). Latent infections often have a low incidence of tumorigenesis and a higher chance of false-negativity as the viral load is too low to be detected using HPV testing. However, nearly 0.05% of HPV-negative cases reportedly progressed to grade 3 CIN (CIN3) or cervical cancer in the subsequent 3–5 years (26).

#### Loss of Human Papillomavirus Fragments During Integration

The HPV L1 fragment is highly conserved in different HPV genotypes; thus, it is targeted by consensus or genotype-specific primers in many HPV detection tests. Integration of the HPV genome into the host genome involves disruption of E1, E2, L2, or L1 fragments (27, 28). HPV testing targeting L1 may be less reliable than that targeting E6/E7 oncogenes, as L1 expression can be lost while E6/E7 expression is always present. The positivity rate of HPV16 and HPV18 detected targeting the HPV L1 region was 91.7% and 72.1%, respectively, suggesting that HPV L1 testing missed 8.3% of HPV16 and 27.9% of HPV18 infections compared with HPV testing targeting E6/E7 (29). However, other researchers believe that HPV L1 testing would rarely result in a false-negative outcome because HPV integration with disruption of L1 coexists with other forms of HPV insertion into the host genome involving disruption of E1/E2 in the same sample.

Studies investigating the mechanisms of HPV infection have demonstrated that a few tumors no longer express the HPV E6/E7 oncogene (HPV-inactive) during cancer development (30). Although the HPV-inactive status is oncogenic, it results in an HPV false-negative outcome using HPV E6/E7 mRNA testing. A study by Banister et al. revealed overall DNA methylation to be decreased, while WNT/β-catenin and Sonic Hedgehog signaling was upregulated in HPV-inactive cervical cancers (30). The somatic mutation profiles differ considerably between HPV-active and HPV-inactive tumors, with more somatic mutations present in HPV-inactive tumors (especially in the TP53, ARID, WNT, and PI3K pathways) (30). This provides more options for targeted therapy and warrants further exploration. Targeting WNT, PI3K, or TP53 mutations may effectively treat HPV-inactive tumors, leading to improved survival outcomes of these patients.

#### Cervical Cancer Caused By Non-High Risk Human Papillomavirus

Several studies have reported the association between cervical cancer and infection with low-risk HPV types 6, 11, 42, 44, and 70 (31–33). Whether low-risk HPV causes cervical cancer or acquired by accident is unknown. Petry et al. estimated that 1%–2% of primary cervical cancers were associated with non-high risk HPV (non-hr-HPV) infection (7), a far higher percentage than the one estimated in the large international collection of invasive cervical cancer (32, 33). Currently, most HPV tests target hr-HPV subtypes and are unable to detect non-hr HPV infection, resulting in partial HPV false-negative results.

#### False-Negative Human Papillomavirus Testing

**Figure 1:**
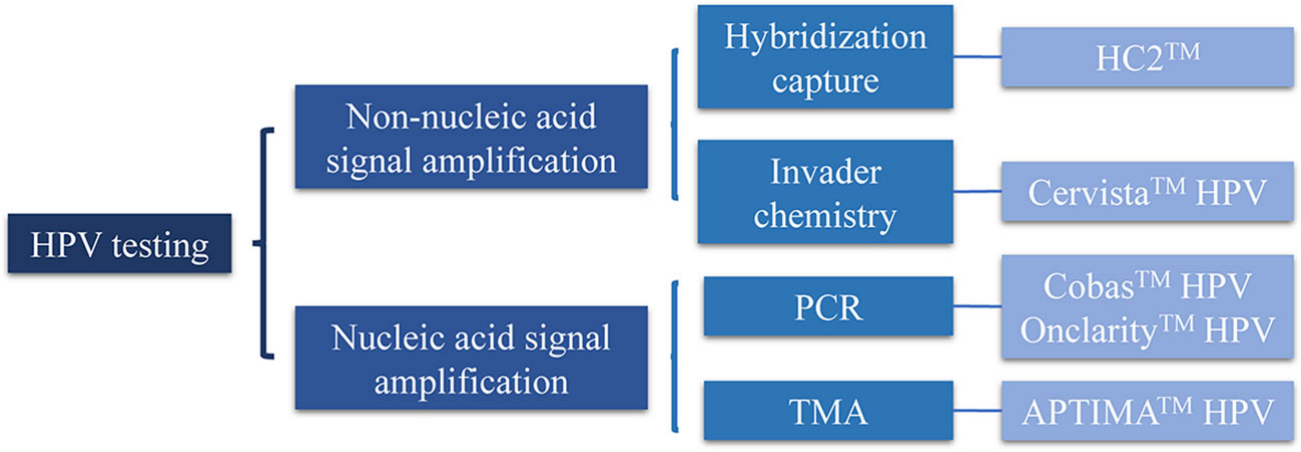
Classification of human papillomavirus (HPV) testing. PCR, polymerase chain reaction; TMA, transcription-mediated amplification.

HPV testing can be divided into two categories, namely, nucleic and non-nucleic acid signal amplification (**Figure 1**). Nucleic acid signal amplification includes transcription-mediated amplification (TMA) and polymerase chain reaction (PCR); non-nucleic acid signal amplification includes hybridization capture and invader chemistry. Currently, the US Food and Drug Administration (FDA) has approved five HPV tests for cervical cancer screening, namely, Hybrid Capture 2™ (HC2™), Cervista™ HPV HR, Cervista™ HPV16/18, Cobas™ HPV, APTIMA™ HPV (28), and BD’s Onclarity™ HPV (**Figure 1**, **Table 1**) (34–50).

**Table 1:** Comparison of FDA-approved human papillomavirus (HPV) testing.

HPV testing(Reference)	Methodology	HPV genotypes	Internal control	Sensitivity CIN2+,%	Specificity CIN2+,%	Analytical Sensitivity
HC2™ (34–39)	Hybrid capture	13 hr-HPV	No	87–98	20–85	1,000–5,000 copies/reaction
Cervista™ HPV HR and HPV16/18 (38, 40–42)	Invader chemistry	14 hr-HPV	Histone 2 gene	90–92.8	44.2–47	625–7,500 copies/reaction for different hr-HPV types
Cobas™ HPV (36, 39, 43–45)	PCR	12 hr-HPV and HPV16/18	β-globin	>88.2	59.3–70.5	150–1,200 copies/ml for different hr-HPV types
APTIMA™ HPV (34, 37, 44, 46)	TMA	14 hr-HPV	Exogenous RNA	87.8–100	>85	17–488 copies/reaction
BD’s Onclarity™ HPV (47–50)	PCR	14 hr-HPV	β-globin	84.6–96.1	46.2–89.1	692–2,990 copies/ml for different HPV types

CIN2+, grade 2 of cervical intraepithelial neoplasia or even worse; HC2, Hybrid Capture 2; hr-HPV, high-risk HPV; PCR, polymerase chain reaction; TMA, transcription-mediated amplification.

Non-nucleic acid signal amplification methods, including HC2™, Cervista™ HPV HR, and Cervista™ HPV16/18, have lower sensitivity but higher cut-off values than nucleic acid signal amplification methods, resulting in false-negative HPV test outcomes, especially when samples have low viral loads. The lack of an internal control in HC2™ also increases the false-negativity rate probably due to DNA contamination or degradation (28, 51). HC2™ covers E1, E2, E4, E5, E6, E7, L1, L2, and LCR fragments of hr-HPV. However, the other three methods targeting L1 alone are prone to false-negative results because of the disruption of L1 fragment during HPV genome integration. APTIMA™ HPV, which differs from HPV DNA testing, detects E6/E7 mRNA of 14 hr-HPV types. Since the expression of E6/E7 mRNA increases after HPV genome integration into the host genome, positive APTIMA™ HPV testing results always indicate cervical cancer or adverse outcomes. It is, therefore, not advisable to use APTIMA™ HPV in primary screening, because E6/E7 mRNA is expressed mainly after HPV integration into the host genome, resulting in a window period between HPV infection and detection. This window phase may increase the rate of HPV false-negative outcomes. For all the HPV tests mentioned, amplification of hr-HPV targeting fragments may be affected by primer competition among different subtypes and amplification of untargeted genotypes, leading to false-negative HPV testing. In conclusion, false-negative HPV testing is associated with lower sensitivity, HPV targeting fragments, hr-HPV genotypes detected, and detection of HPV DNA or RNA.

In addition to the method used, HPV false-negative results are also related to sampling errors. Poor cell viability from necrotic and/or inflammatory sites often results in HPV false-negative outcomes. Faulty sample collection methods, including samples mixed with blood or lubricant, as well as fixation procedure can result in false negativity. Therefore, the accuracy of HPV testing in published studies should be questioned discreetly. Some studies employed HPV testing to investigate previously stored cervical cancer specimens, but it is unknown whether such specimens precisely reflect HPV infection in the patients. A retrospective study demonstrated that samples from elderly patients or those stored for a longer duration had lower HPV-positivity rates (13). The effect of storage time on HPV positivity was more distinct in adenocarcinoma than in squamous cell carcinoma (13). Other factors that affect HPV positivity include the time between excision to fixation and fixator type (13). In a retrospective study, the use of unbuffered formalin fixation was an important factor influencing HPV-negative results (13).

In summary, the most important reason for false-negative HPV testing results is the significant difference among HPV detection methods, which is not realized by all clinicians (29). Testing procedures and sample quality, as in DNA/RNA degradation of formalin-fixed and paraffin-embedded samples, can also lead to HPV false-negative results.

## Clinical Features of Human Papillomavirus-Negative Cervical Cancer

### Age

A global study involving 760 cases of cervical adenocarcinoma revealed that older patient age at initial diagnosis was associated with a lower positivity rate of HPV DNA testing (13). A similar trend was identified in squamous cell carcinoma without any clear reason. One possible explanation is that viral vitality is gradually lost during tumor progression, especially in older patients with more time to develop cancer. Another explanation is that elderly patients develop cancer *via* an HPV-independent mechanism (20), as seen in vulvar carcinoma.

### Molecular and Pathological Features

Pathological type influences the results of HPV detection, as demonstrated by differences in HPV infection rates between cervical squamous cell carcinoma and adenocarcinoma. Globally, 12.7% of squamous cell carcinoma and 15%–38% of cervical adenocarcinoma are HPV-negative (10, 19). The parakeratosis or hyperkeratosis status of squamous cell carcinoma can lead to false-negative HPV testing results (52). Additionally, HPV-negative cervical cancer whose histopathology is almost all adenocarcinoma is possibly missed by HPV testing (8, 18, 53). The positivity rate of some HPV genotypes in cervical adenocarcinoma was reportedly low (17). Moreover, the HPV DNA load in cervical adenocarcinoma was lower than that in squamous cell carcinoma, challenging HPV detection in adenocarcinoma (17). The glandular epithelium is not susceptible to persistent HPV infection; accumulation of free HPV DNA as well as copy numbers of integrated HPV were low even in HPV-infected glandular epithelium (13). In contrast, HPV-infected squamous cell carcinoma tended to have higher copy numbers of HPV DNA and integrated virus.

The common pathological types of HPV-positive adenocarcinoma are intestinal, villoglandular, signet-ring cell, and endometrioid adenocarcinoma, which originates from the cervical squamous columnar junction zone, accounting for nearly 90% of all cervical adenocarcinomas (**Table 2**) (13–15, 17, 18, 54). The pathological types of HPV-negative adenocarcinoma are gastric, clear cell, serous, and mesonephric adenocarcinomas (**Table 2**). These types are quite rare and their occurrence might not be HPV-related (13).

**Table 2:** Pathological types of cervical adenocarcinoma and its human papillomavirus (HPV)-positive rate.

Pathological types (Reference)	Percentage of cervical adenocarcinoma, %	HPV-positive rate, % (17)
Endocervical (usual) type (15, 18)	73–79	80–100
Intestinal (15)	3–8	83–100
Villoglandular (15)	0.8–6	100
Signet-ring cell (15)	0.3	100
Endometrioid (13, 15)	1.1–1.6	27.3
-From squamous columnar junction zone	—	100
-From upper endocervix and lower uterine segment	—	0
Gastric (15, 18)	1.5–10	0
Clear cell (13, 18)	4.4–6.3	20–27.6
Serous (13, 15)	0.5–3.5	25–30.4
Mesonephric (15)	0.3	0

The pathogenesis of these HPV-independent pathological types is correlated with specific mutations of the genome. The PI3K-AKT pathway may be involved in the development of clear cell adenocarcinoma; immunostaining results for p-AKT and p-mTOR were positive in 50% of cases (17, 55). In elderly patients with this adenocarcinoma subtype, PTEN expression was lost in 50% of patients, while EGFR and HER2 expression increased in 75% and 50% of patients, respectively (17, 55). Gastric adenocarcinoma has been associated with somatic and germline mutations of STK11 and TP53 (Peutz-Jeghers Syndrome) (14). PIK3CA, PTEN, and CTNNB1 mutations have been frequently reported for endometrioid adenocarcinoma (20). In mesonephric adenocarcinoma, 81% of patients harbored KRAS or NRAS mutations, while 62% carried mutations of ARID1A, ARID1B, or SMARCA4, but none of PIK3CA or PTEN (17, 56). The characteristic mutation of mesonephric adenocarcinoma differed from the common mutations of cervical adenocarcinoma, with 7% of cases harboring KRAS/NRAS mutations (56). Therefore, RAS/MAPK pathway inhibitors may provide potential treatment options for mesonephric adenocarcinoma.

### FIGO Stage

HPV-negative patients are prone to develop advanced FIGO stage and lymphatic space invasion prior to diagnosis, resulting in poor prognosis (**Table 3**) (57, 58). A multicenter study revealed that 62.5% of HPV-negative adenocarcinomas were stage II or higher, while 83.7% of usual type cases were stage I at diagnosis, which concurred with previous studies (60). Further, HPV-negative cases in this study exhibited a larger tumor size than HPV-positive cases.

**Table 3:** Studies of FIGO stage and prognosis of human papillomavirus (HPV)-negative cervical cancers.

Study (Reference)	Cases (HPV negative/overall)	HPV testing	Advanced FIGO stage (HPV negative vs. HPV positive)	Lymphatic metastasis (HPV negative vs. HPV positive)	DFS (HPV negative vs. HPV positive)	OS (HPV negative vs. HPV positive)
Nicolas et al. (57)	21/214	PCR	91% vs. 57%, *p*<0.01	67% vs. 36%, *p*<0.01	59.8 m (95%CI 32.0–87.6 m) vs. 132.2 m (95%CI 118.6–145.8 m), *p*<0.01	77.0 m (95%CI 47.2–106.8 m) vs. 153.8 m (95%CI 142.0–165.6 m), *p*=0.01
Van der Marel et al. (58)	8/136	HC2™, PCR L1&E7	87.5% vs. 52.3%, *p*=0.053	37.5% vs. 17.2%, *p*=0.150	51.9 m (95%CI 12.2–91.7 m) vs. 109.9 m (95%CI 98.2–121.5 m), *p*=0.010	67.7 m (95%CI 20.0–106.9 m) vs. 108.9 m (95%CI 97.7–120.0 m), *p*=0.225
Feng et al. (59)	43/122	PCR (HPV 16/18)	—	—	—	5 year: HR=1.250 (95%CI 0.562–2.784), *p*=0.584 8 year: HR=1.530 (95%CI 0.697–3.362), *p*=0.289

PCR, polymerase chain reaction; HC2, Hybrid Capture 2; DFS, disease-free survival; OS, overall survival; m, months; HR, hazard ratio; 95%CI, 95% confidence interval.

## Treatment of Human Papillomavirus-Negative Cervical Cancer

Currently, HPV-negative cervical cancer has no specific therapy and thus consults with HPV-positive cervical cancer treatment strategies. Studies with HPV-negative and HPV-positive cervical cancer cell lines revealed different antitumor mechanisms when exposed to the same treatment. For example, a histone deacetylase (HDAC) inhibitor repressed E6 activity to promote apoptosis in HPV-positive cervical cancer cells but caused G2 phase arrest in HPV-negative cervical cancer cells, while dehydroepiandrosterone caused apoptosis in HPV-positive cervical cancer cells and necrosis in HPV-negative cervical cancer cells (61, 62). Based on the etiology of HPV-related cervical cancer, gene expression in 74 cell lines demonstrated significantly higher p16 expression while that of phosphorylated retinoblastoma protein (pRb) was lower in HPV-positive cell lines, compared with HPV-negative cell lines (63). Abemaciclib, a CDK4/6 inhibitor, suppressed CDK4/6-Rb-E2F and mTOR pathways, resulting in superior treatment in HPV-negative cancer (64). The overall survival (OS) rate of patients who received surgery combined with other oncologic treatment differed significantly between HPV-positive and HPV-negative cases, while that of patients who received surgery alone did not (60), suggesting that adjuvant chemoradiotherapy may benefit HPV-negative cases. True HPV-negative cervical cancers are associated with specific pathological types, therefore understanding their tumorigenesis will contribute to the selection of suitable therapies for cervical cancer. Much attention has focused on exploring TP53, ARID, WNT, and PI3K pathways, which mutate frequently in cervical adenocarcinoma, to develop effective targeted therapies. Recently, lncRNA has emerged as a research hotspot for HPV-negative cervical cancer treatment (65, 66).

## Prognosis of Human Papillomavirus-Negative Cervical Cancer

As early as 1990, a study of 106 early-stage invasive cervical cancer cases using PCR revealed that the risk of overall relapse did not differ among different HPV genotypes in HPV-positive patients, but was 2.6 times higher in HPV-negative patients and their risk of distant metastasis was 4.5 times higher than HPV-positive patients. The 24-month relapse-free survival rate of HPV-positive patients was higher than that of HPV-negative patients (77% vs. 40%) (67). A meta-analysis of 2838 cervical cancer cases from 17 studies revealed that HPV positivity correlated with better prognosis (OS: HR=0.610, *p*=0.001; disease-free survival: HR=0.362, *p*<0.001) *(* 68). To date, three other studies have reached the same conclusion that HPV-negative cervical cancer is associated with poor prognosis (**Table 3**) (57–59). Nevertheless, a 10-year follow-up study of 204 patients with cervical cancer revealed that the 5-year OS rate of HPV-negative and HPV-positive patients was 82% and 58% (*p*=0.003), respectively, indicating that HPV infection was significantly correlated with poor OS of patients (69). Further investigation is warranted to elucidate the effects of negative HPV testing on prognosis.

## Discussion

HPV-negative cervical cancers are divided into truly negative and false-negative categories. Truly negative cervical cancers have an HPV-independent pathogenesis with specific pathological types, of which HPV vaccination and testing probably have little effect on their prevention. The diagnosis of truly negative cervical cancer mainly depends on cytological screening and observation of histological features, combined with cytological multiple staining. Further investigation of the pathways and biomarkers of the different pathological types is required to develop a basis for precise therapy. For false-negative cervical cancer, retesting should be considered using other HPV testing methods according to their characteristics after analyzing the reason for false-negative HPV results. HPV-positive results after retesting may be due to initial testing failures for hr-HPV or the inability of the standard HPV test to detect other HPV genotypes (7). Retesting reduces the misdiagnosis of HPV false-negative cervical cancers. Most HPV-negative samples from reported studies were formalin-fixed and paraffin-embedded, which affects HPV DNA quality and causes false-negative results. Improving HPV detection strategies by developing standardized and high-quality HPV tests is crucial to reduce false negativity. Different procedures for sample collection, storage, and testing affect HPV test outcomes, therefore operating procedures for HPV testing need to be standardized. It is beneficial to choose more sensitive HPV testing verified by universal standards and to consider the cut-off values of HPV testing, especially in persistent HPV infections with low viral activity (70). Further, the laboratory conducting HPV testing should be authorized by institutions and meet international standards (71).

Cervical adenocarcinoma is the major pathological type of HPV-negative cervical cancer, most likely caused by mutations of PI3K-AKT or other pathways. Preclinical studies have demonstrated that tumorigenesis differs between HPV-positive and HPV-negative cervical cancers, which presents the possibility of developing targeted therapies for HPV-negative patients. This may provide a basis for cervical cancer treatment research in the future. Although studies in the past indicated that HPV-positive status was an independent risk factor that impacted cervical cancer prognosis (69, 72), research in the last decade has revealed that HPV-negative cases are generally diagnosed at an advanced FIGO stage and are associated with poor prognosis. Large-scale multicenter studies need to be conducted to further elucidate the relationship between HPV negativity and cervical cancer.

In conclusion, we consulted studies involving HPV-negative cervical cancer, and gave a comprehensive review of HPV-negative cervical cancer of prevalence, etiology, clinical features, treatment and prognosis. Although HPV-negative cervical cancer reveals different characteristics from HPV-positive one, most studies ignore the HPV status of cervical cancer, which restricts a profound insights of HPV-negative cervical cancer. Clarifying the different categories of HPV-negative cervical cancers is crucial to the development of suitable treatments and to guide studies investigating HPV-negative cervical cancers. The presence of HPV-independent cervical cancers should not affect the promotion of HPV testing and vaccination. Clinicians should classify and treat HPV-negative cases cautiously, and consider the correlation between advanced stage and poor prognosis of cervical cancer to provide women with negative HPV testing better management and effective treatment.

## Author Contributions

YZ and GW provided the concept and designed the study. BX and JG wrote the draft of the manuscript. YZ and YS gave critical revision of the manuscript. YZ and JG provided economic support to the study. The authors meet all the criteria set out in the journal’s authorship criteria. All authors contributed to the article and approved the submitted version.

## Funding

This study was funded by the National Natural Science Foundation of China (Grant Nos. 81974463 and 81672573).

## Conflict of Interest

The authors declare that the research was conducted in the absence of any commercial or financial relationships that could be construed as a potential conflict of interest.
